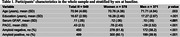# Sex and Age Differences in the Association of Serum GFAP Levels with Cognitive Decline in Cognitively Unimpaired Individuals: Results from the A4 Study

**DOI:** 10.1002/alz70856_103439

**Published:** 2025-12-24

**Authors:** Elham Ghanbarian, Babak Khorsand, Laura A. Rabin, Richard B. Lipton, Seyed Ahmad Sajjadi, Ali Ezzati

**Affiliations:** ^1^ University of California, Irvine, Irvine, CA, USA; ^2^ Brooklyn College of the City University of New York, Brooklyn, NY, USA; ^3^ Albert Einstein College of Medicine, Bronx, NY, USA

## Abstract

**Background:**

Inflammation plays a central role in Alzheimer's Disease (AD) pathology and may contribute to sex differences in AD risk. Elevated levels of glial fibrillary acidic protein (GFAP), a marker of astrocytic reactivity, have been associated with faster rates of cognitive decline. However, its role in sex‐specific cognitive decline trajectories in AD is not well understood. We investigated sex differences in the association between GFAP levels and cognitive decline in cognitively unimpaired (CU) individuals.

**Method:**

For the cross‐sectional analysis, we included 949 CU participants with serum GFAP levels from the A4 (*n* = 499, amyloid positive) and the LEARN study (*n* =  450, amyloid negative). Linear regression models were used to assess the association between GFAP and the Preclinical Alzheimer Cognitive Composite (PACC), the primary A4 study outcome measure, cross‐sectionally. The longitudinal analysis included 695 participants (A4 placebo arm: *n* = 245; LEARN: *n* = 450). Linear mixed‐effect models estimated PACC changes over 240 weeks based on the baseline GFAP levels, stratified by sex and age groups (65–75 vs. >75 years).

**Result:**

At baseline, participants were on average 70.94±4.69 years old, and GFAP levels were higher in women compared to men (0.12±.06 vs 0.10±.05, *p* <.001, Table 1). GFAP levels were negatively associated with PACC in women (*n* =  578, ß=‐4.58, *p* = .006) but no significant association was observed in men (*n* =  371, ß=‐3.33, *p* = .15). Longitudinally, higher baseline GFAP levels were significantly associated with higher rates of decline in PACC scores in women (ß=‐6.08, *p* = .001), but not in men (ß=‐3.97, *p* = .08). When participants were categorized into younger (65‐75 years old, *n* = 573) vs. older (> 75, *n* = 122) groups, GFAP levels were associated with PACC in both age groups in women (younger: *n* = 351, ß=‐6.54, *p* = .003; older: *n* = 67, ß=‐9.63, *p* = .016), but only in the older group of men (*n* = 55, ß=‐7.45, *p* = .022). When participants were categorized into amyloid positive and negative groups (A4 vs. LEARN participants), GFAP levels were negatively associated with PACC only in amyloid negative women (*n* = 278, ß=‐8.50, *p* <.001).

**Conclusion:**

These findings suggest that serum GFAP may serve as a biomarker for identifying CU women at greater risk of cognitive decline, underscoring the need for sex‐specific strategies in AD risk assessment and prevention.